# Teleoperation System for Service Robots Using a Virtual Reality Headset and 3D Pose Estimation

**DOI:** 10.3390/s26020471

**Published:** 2026-01-10

**Authors:** Tiago Ribeiro, Eduardo Fernandes, António Ribeiro, Carolina Lopes, Fernando Ribeiro, Gil Lopes

**Affiliations:** 1 Automation and Robotics Laboratory, Algoritmi Center, University of Minho, 4800-058 Guimaraes, Portugal; id9402@alunos.uminho.pt (T.R.); eacfernandes1999@gmail.com (E.F.); id11906@alunos.uminho.pt (A.R.); pg50176@alunos.uminho.pt (C.L.); fernando@dei.uminho.pt (F.R.); 2 LIACC—Laboratory of Artificial Intelligence and Computer Science, Polytechnic Institute of Porto, 4200-072 Porto, Portugal

**Keywords:** human–robot interaction, teleoperation, virtual reality, 3D pose estimation, RGB-D sensing, service robots

## Abstract

This paper presents an immersive teleoperation framework for service robots that combines real-time 3D human pose estimation with a Virtual Reality (VR) interface to support intuitive, natural robot control. The operator is tracked using MediaPipe for 2D landmark detection and an Intel RealSense D455 RGB-D (Red-Green-Blue plus Depth) camera for depth acquisition, enabling 3D reconstruction of key joints. Joint angles are computed using efficient vector operations and mapped to the kinematic constraints of an anthropomorphic arm on the CHARMIE service robot. A VR-based telepresence interface provides stereoscopic video and head-motion-based view control to improve situational awareness during manipulation tasks. Experiments in real-world object grasping demonstrate reliable arm teleoperation and effective telepresence; however, vision-only estimation remains limited for axial rotations (e.g., elbow and wrist yaw), particularly under occlusions and unfavorable viewpoints. The proposed system provides a practical pathway toward low-cost, sensor-driven, immersive human–robot interaction for service robotics in dynamic environments.

## 1. Introduction

Service robots are increasingly employed in various domains, including assistance, education, and entertainment, requiring reliable and efficient remote-control mechanisms. A fundamental aspect of these systems lies in their ability to interpret human commands—whether via traditional interfaces or gesture-based control. Despite the growth of the service robotics sector, the definition of a “service robot” remains somewhat ambiguous. However, the International Federation of Robotics (IFR) defines service robots as semi- or fully autonomous machines designed to provide services that improve human well-being, explicitly excluding industrial manufacturing applications [1,2].

As these robots are deployed in increasingly complex and safety-critical environments, full autonomy may be impractical or undesirable. In such scenarios, teleoperation—where a human operator remotely controls the robot—is a key enabling technology [3]. Teleoperation allows humans to extend their perception and manipulation capabilities beyond immediate physical boundaries through communication links that bridge the human operator and the robot’s sensors and actuators [4].

Teleoperation paradigms can be categorized into three main types, depending on the degree of human intervention: direct, coordinated, and task-based [3]. In direct teleoperation, the user controls the actuators in real time with continuous feedback, as in the case of remote-controlled vehicles. Coordinated teleoperation introduces an assistance layer, enabling control through high-level parameters such as speed or orientation. Task-based teleoperation allows partial autonomy, with the operator providing strategic-level commands through human–machine interfaces (HMIs), while the robot handles execution.

One of the key technologies enabling intuitive teleoperation is human pose estimation—the process of determining the spatial configuration of body joints from visual data, including landmarks of the face, hands, and limbs. Combined with Virtual Reality (VR), pose estimation offers immersive and natural interfaces for remote operation. VR systems, comprising head-mounted displays (HMDs), motion tracking devices, and real-time rendered environments, allow users to interact intuitively with the robot, enhancing task engagement and situational awareness [5,6,7].

This work is conducted within the scope of the CHARMIE project (Collaborative Home/Healthcare Assistant Robot by Minho Industrial Electronics) [8], developed at the Laboratory of Automation and Robotics, University of Minho. The primary goal is to design and implement an immersive teleoperation framework that enables effective control of the CHARMIE service robot. The proposed system transmits sensory data—including camera feeds and sensor outputs—into a VR environment. Simultaneously, a pose estimation pipeline processes the operator’s body movements and maps them onto the robot’s kinematic structure, enabling tasks such as object manipulation. This approach establishes a seamless link between human intention and robotic execution, paving the way for advanced, human-centered service robotics

## 2. Related Work

Recent advancements in VR technologies have significantly influenced the development of teleoperation systems for service robots, enabling more natural, immersive, and efficient human–robot interactions. The convergence of VR with robotics has facilitated new paradigms of control, particularly in tasks requiring precision, adaptability, and intuitive manipulation in complex or remote environments.

One of the earliest influential contributions in this field was proposed by researchers at MIT’s Computer Science and Artificial Intelligence Laboratory (CSAIL), who introduced a VR interface based on the concept of a homunculus model. Their system immersed the human operator within the robot’s control space using head-mounted displays, allowing real-time visual feedback and intuitive manipulation of robotic limbs. The results indicated a marked improvement in task performance compared to conventional control methods, particularly in scenarios involving object manipulation [9].

In parallel, other frameworks have aimed to broaden the scope of VR-based teleoperation by integrating multiple dimensions of interaction. A notable example is the five-dimensional teleoperation architecture proposed in the literature, encompassing control, visualization, interaction, usability, and infrastructure. This holistic approach sought to address the limitations of traditional teleoperation systems by enhancing adaptability and user experience, allowing complex tasks to be executed with minimal operator training [10].

The industrial robotics domain has also explored the application of VR interfaces for remote manipulator control. Studies in this area identified critical challenges, particularly regarding limited access to low-level motion parameters such as joint torques or actuator velocities. To address these constraints, researchers proposed signal processing techniques—such as filtered command smoothing—to enhance stability and responsiveness during fine manipulation, thereby improving system usability under real-world constraints [11].

Complementing visual immersion with tactile perception, recent developments have introduced bimanual telerobotic systems incorporating haptic feedback. These systems integrate dual-arm configurations and dexterous effectors with force-reflective devices, enabling the operator to receive tactile cues in addition to visual feedback. Empirical evaluations demonstrated that haptic feedback significantly enhances depth perception, task accuracy, and reduces cognitive load, particularly in precision-critical applications [12].

Recent research on humanoid robot teleoperation has increasingly focused on motion imitation and whole-body control to achieve more natural and intuitive human–robot interaction. Vision-based and motion-capture-driven approaches have been widely explored to map human kinematics onto humanoid platforms while preserving stability and responsiveness. For example, Sripada et al. proposed a teleoperation framework based on RGB-D sensing that enables real-time upper-body imitation combined with basic legged locomotion, allowing a humanoid robot to walk, turn, and replicate human arm motions with minimal latency. Their work highlights the feasibility of low-cost, vision-based teleoperation but also reveals limitations related to sensor noise, restricted viewpoints, and simplified motion mapping strategies [13].

More immersive and expressive teleoperation systems have been demonstrated using full-body motion capture and virtual reality interfaces. Wang et al. presented a full-body teleoperation architecture in which a humanoid robot is controlled through a motion-capture suit and a VR headset, providing first-person telepresence and intuitive whole-body imitation. Their results show that immersive telepresence and high-dimensional motion mapping can significantly reduce operator training time and improve task versatility. However, such systems typically rely on specialized hardware, increasing system cost and limiting accessibility [14].

Motion retargeting and expressive control have also been addressed through optimization-based approaches. Mohan and Kuchenbecker introduced a real-time kinematic retargeting method that emphasizes whole-arm expressiveness rather than strict end-effector tracking, enabling more human-like robot motion during teleoperation. While these approaches improve motion naturalness and adaptability across different robot morphologies, they often focus on upper-body control and assume accurate motion capture inputs. In contrast, the present work focuses on a lightweight, vision-based teleoperation pipeline that prioritizes simplicity, deployability, and real-time operation, while acknowledging the trade-offs in precision inherent to non-contact sensing [15].

Collectively, these studies underscore the growing maturity of VR-based teleoperation, emphasizing the importance of multimodal feedback, immersive interfaces, and adaptive system architectures. The integration of VR not only enhances the operational capabilities of service robots but also opens pathways for their effective deployment in diverse and dynamic environments.

Despite significant advances in VR-based teleoperation and vision-based human pose estimation, several limitations remain in existing systems. Many approaches rely on high-cost motion capture setups or instrumented environments, limiting their applicability in domestic or service robotics contexts. Other works focus primarily on simulation or virtual avatars, without validation on real robotic platforms operating under physical constraints. Additionally, vision-only pose estimation pipelines often struggle with axial joint rotations and occlusions, which are rarely analyzed in depth or explicitly acknowledged in experimental evaluations.

Motivated by these limitations, this work aims to develop a low-cost and reproducible teleoperation framework that integrates consumer-grade VR hardware, RGB-D sensing, and real-time pose estimation, while being validated on a physical service robot. Rather than seeking perfect joint-level reconstruction, the proposed approach prioritizes functional task execution and system robustness under realistic operating conditions typical of service robotics environments.

The main contributions of this work can be summarized as follows:

- A low-cost immersive teleoperation framework that combines a virtual reality interface with real-time 3D human pose estimation using consumer-grade hardware.
- A practical RGB-D–based pipeline for reconstructing 3D human joint positions and mapping them to the kinematic constraints of an anthropomorphic service robot arm.
- Geometric strategies for joint angle estimation, including explicit handling and analysis of axial rotation limitations in elbow and wrist joints.
- Experimental validation on a real service robot, demonstrating reliable object manipulation through both direct and VR-based teleoperation.
- A critical discussion of system limitations, providing transparent insight into the challenges of vision-only teleoperation and motivating concrete future extensions.

## 3. Materials and Methods

Human pose estimation in three-dimensional space has gained considerable attention due to its relevance in applications such as human–robot interaction, virtual reality, and motion analysis. Although significant progress has been made in recent years, pose estimation remains a challenging task, especially when precise spatial mapping is required for robotic control in real time.

System Overview

**Figure 1:**
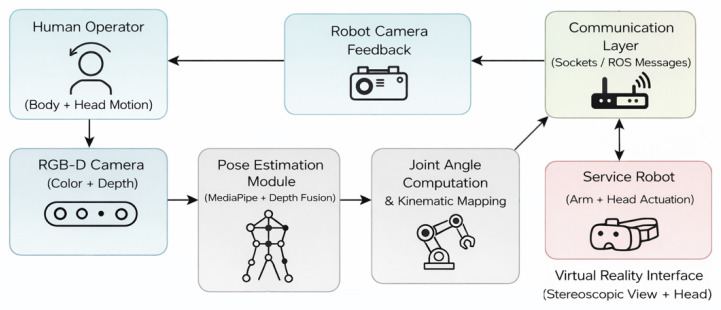
High-level block diagram of the proposed VR-based teleoperation system.

The proposed teleoperation system follows a modular architecture that integrates human motion capture, real-time processing, robotic actuation, and immersive feedback. Figure 1 presents a high-level block diagram of the system, highlighting the main functional components and data flows.

Human motion is captured using an RGB-D camera, providing synchronized color and depth information. A pose estimation module extracts body landmarks and reconstructs the operator’s 3D joint configuration. Joint angles are then computed and mapped to the kinematic constraints of the service robot’s anthropomorphic arm. Control commands are transmitted to the robot through a dedicated communication layer, while visual feedback from the robot-mounted camera is streamed back to the operator. A virtual reality interface provides stereoscopic visualization and head-motion-based view control, closing the teleoperation loop. This modular structure enables clear separation between sensing, processing, communication, and actuation, facilitating system integration and future extensions.

Communication between the processing modules and the robotic platform is implemented through a lightweight message-based architecture. Joint angle commands and head orientation data are transmitted from the processing unit to the robot controller using network sockets, while sensor and camera data are streamed in the opposite direction. This approach enables modular separation between perception, control, and actuation components, while maintaining sufficient responsiveness for real-time teleoperation in indoor environments. The communication layer was designed to be hardware-agnostic, allowing integration with microcontroller-based platforms and middleware such as ROS, depending on deployment requirements.

In the proposed system, human pose estimation is achieved through Google’s open-source MediaPipe framework (version 0.8.10) [16], which enables simultaneous detection of full-body landmarks, facial features, and hand positions. MediaPipe employs a holistic approach that integrates individual models into a single processing pipeline, allowing for efficient and unified inference of 33 key landmarks across the human body.

To control the robot based on the detected human posture, a systematic pipeline is employed. Initially, MediaPipe extracts the 2D coordinates of key anatomical points (e.g., shoulders, elbows, wrists), to which a depth component is later added to construct a 3D model of the operator’s pose. The angular configuration of each human joint is then computed using vector operations—such as dot and cross products—allowing for the extraction of joint angles, including shoulder pitch or elbow flexion. These angles are subsequently mapped to the corresponding robot joints through linear scaling or proportional mappings, ensuring that the robot’s motion adheres to its physical joint constraints while reflecting the operator’s movements as closely as possible.

Given that MediaPipe natively operates in a 2D coordinate space, an RGB-D (Red-Green-Blue plus Depth) sensor is incorporated to extend the spatial representation into three dimensions. Each key point is thus enriched with depth information (z-coordinate), enabling accurate 3D reconstruction of the human pose. To evaluate depth sensing performance, a comparative analysis was conducted using two RGB-D cameras: the Intel^®^ RealSense™ D455 and the Microsoft Kinect for Xbox 360. The Intel sensor provides color-coded depth maps, while the Kinect device utilizes a grayscale depth encoding, where darker shades correspond to greater distances. Table 1 shows the technical specifications of both cameras.

**Figure 2:**
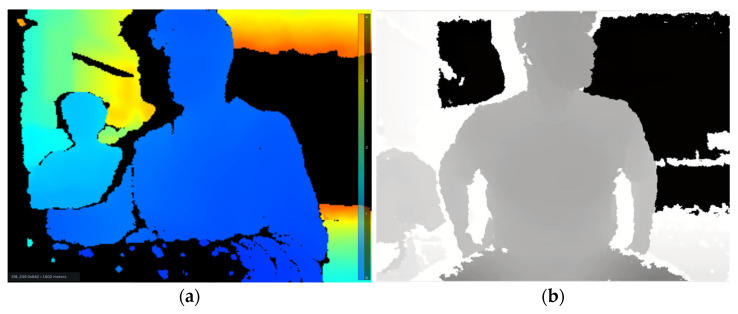
Intel® RealSense™ D455 (**a**) and Microsoft Kinect XBOX 360 (**b**) depth maps.

As illustrated in Figure 2, the two systems differ in precision and visual representation, affecting the fidelity of joint position estimation and, consequently, the accuracy of robot motion control.

**Figure 3:**
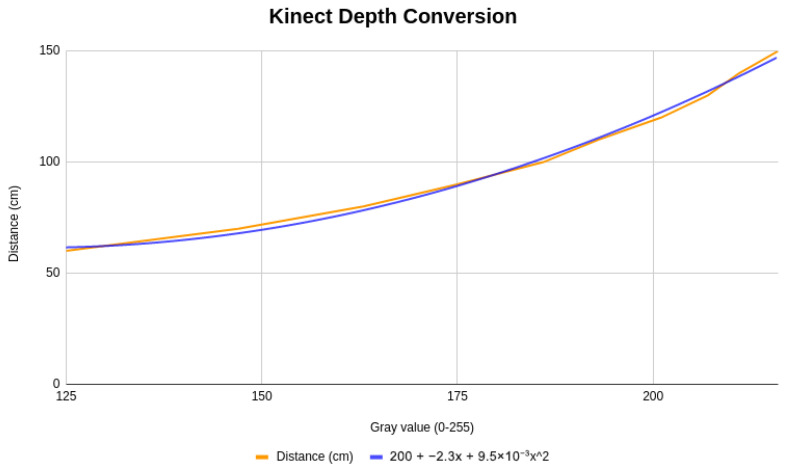
Microsoft Kinect XBOX 360: depth conversion from grayscale to distance.

Among the evaluated devices, the Intel^®^ RealSense™ D455 offers a built-in method, get_distance(), which provides direct access to depth measurements in meters. This functionality simplifies the acquisition of reliable depth data, streamlining the mapping process between human and robot kinematics. In contrast, the Microsoft Kinect requires a more elaborate calibration procedure. Depth information must be inferred by converting grayscale intensity values into metric distances, which involves collecting reference measurements at multiple points and fitting a calibration curve. This calibration process is illustrated in Figure 3, which shows the mapping between grayscale pixel intensity and real-world distance for the Microsoft Kinect system.

Although this mapping enables approximate depth estimation using a monocular grayscale representation, it is inherently sensitive to sensor noise, lighting conditions, surface reflectivity, and viewpoint variations. Small deviations in pixel intensity can translate into non-negligible distance errors, particularly at larger depths, where the calibration curve becomes less linear. These effects directly influence the accuracy of joint reconstruction and propagate to the estimation of joint angles, especially for rotations around the limb axis. Consequently, the calibration illustrated in Figure 3 highlights a fundamental trade-off of vision-based teleoperation: reduced hardware complexity and cost are achieved at the expense of depth precision. In the proposed system, this limitation is mitigated at the task level through continuous visual feedback and human-in-the-loop correction, which proved sufficient for stable teleoperation in manipulation scenarios.

A comparative analysis between the Intel^®^ RealSense™ D455 and the Microsoft Kinect Xbox 360 was carried out to assess their effectiveness in acquiring depth information for human pose estimation within the proposed teleoperation system. The RealSense D455 demonstrated superior performance, offering depth accuracy with typical errors under 2% at distances up to 6 m. Additionally, its higher depth resolution allows for finer capture of human joint details, which is critical for precise motion tracking. In contrast, the Kinect Xbox 360, though more accessible and supported by a broad developer community, exhibits lower depth resolution and reduced accuracy at greater ranges. These limitations can compromise the reliability of pose estimation, particularly for complex gestures. The RealSense D455 also benefits from advanced SDK capabilities and built-in RGB-D support, which facilitate its integration with the MediaPipe framework. For these reasons, the D455 was selected as the depth sensor for the system, offering improved pose fidelity and overall system robustness.

To accurately compute joint movements and resolve the degrees of freedom involved, a dedicated Python (version 3.8)-based algorithm was implemented. The MediaPipe library is used to identify key body landmarks in the 2D RGB stream. Each point’s 3D coordinate is then reconstructed by querying its corresponding depth value using the RealSense get_distance() function. This enables the creation of a 3D skeletal representation of the user.

Joint angles are then calculated using vector operations. Specifically, the angle *θ* between two vectors *A* and *B*, sharing a common origin, is determined using the scalar (dot) product, defined as:

(1) $$\theta =\cos^{-1}\left[\frac{A\cdot B}{∥A∥∥B∥}\right],$$

Here, *A* and *B* denote the dot product of vectors *A* and *B*, and ||*A*|| and ||*B*|| represent their respective Euclidean norms. This formulation provides a reliable and computationally efficient method for angle calculation between limb segments.

The introduction of depth data, while enhancing precision, also presents challenges such as joint occlusion and overlapping points, which may degrade estimation quality. To mitigate this, a rotatable array structure is proposed to temporarily store successive joint readings. By rotating and averaging these values, overlapping can be accounted for, improving the stability of depth estimation.

**Figure 4:**
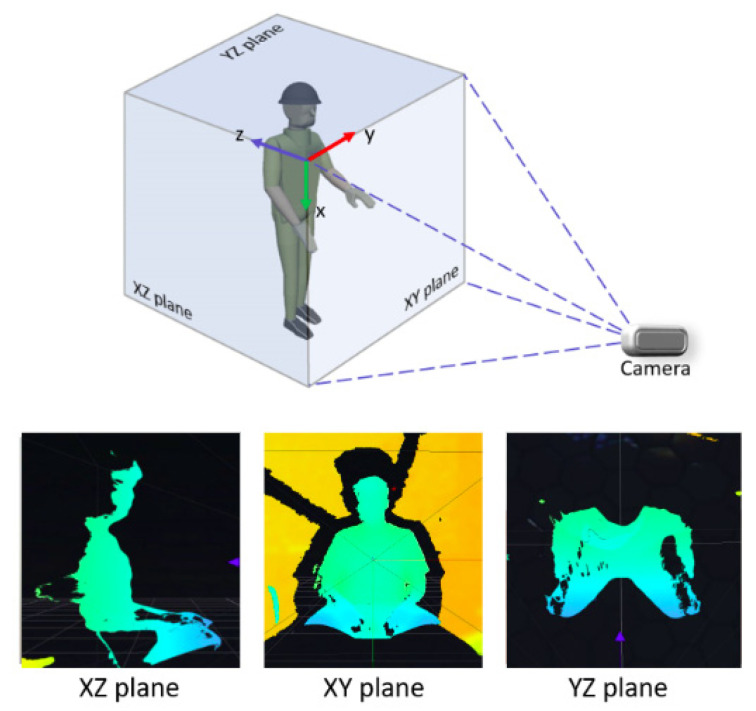
Orthogonal projection of the 3D view.

In certain scenarios, full 3D computation may not be necessary. For example, shoulder pitch can often be resolved within a 2D projection. To streamline computations, the 3D coordinates of joints can be decomposed into orthogonal planes—XY (frontal), YZ (lateral), and XZ (top)—allowing targeted analysis of specific movements. This projection-based strategy, illustrated in Figure 4, reduces computational load while maintaining sufficient accuracy for selected degrees of freedom. In these visualizations, the color scale encodes the depth value per pixel, mapping each point’s distance to the camera within the captured RGB-D scene.

Shoulder Pitch Calculation

**Figure 5:**
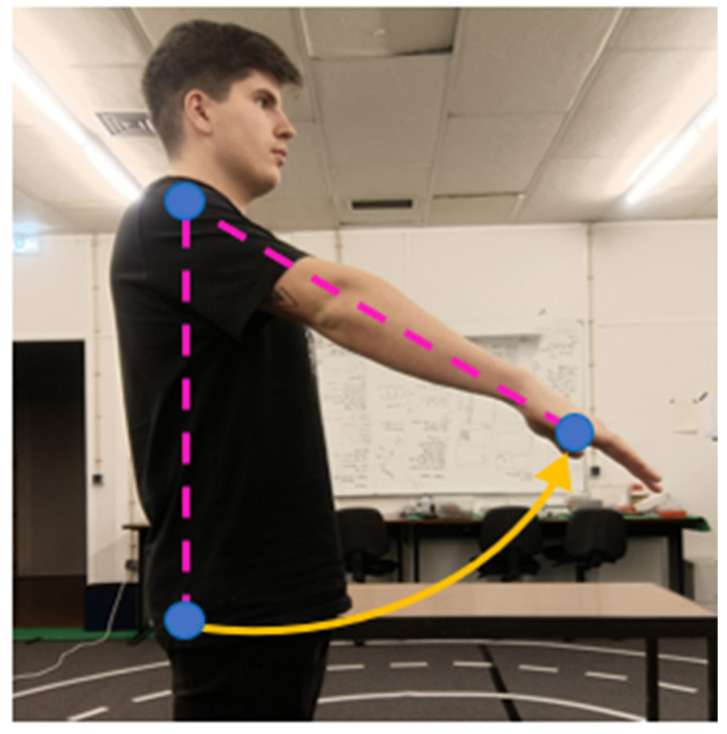
Representation of shoulder pitch using lateral projection (x–z plane).

Shoulder pitch refers to the rotational motion of the shoulder in the sagittal plane, corresponding to the forward–backward movement of the upper arm along the *Y*-axis. To compute this degree of freedom, three key points are used: hip, shoulder, and elbow. Since the movement occurs in the lateral plane, only the x and z coordinates are considered in the scalar product calculation. The resulting angle represents the arm’s inclination relative to the torso in the sagittal plane. This approach is illustrated in Figure 5.

Shoulder Roll Calculation

**Figure 6:**
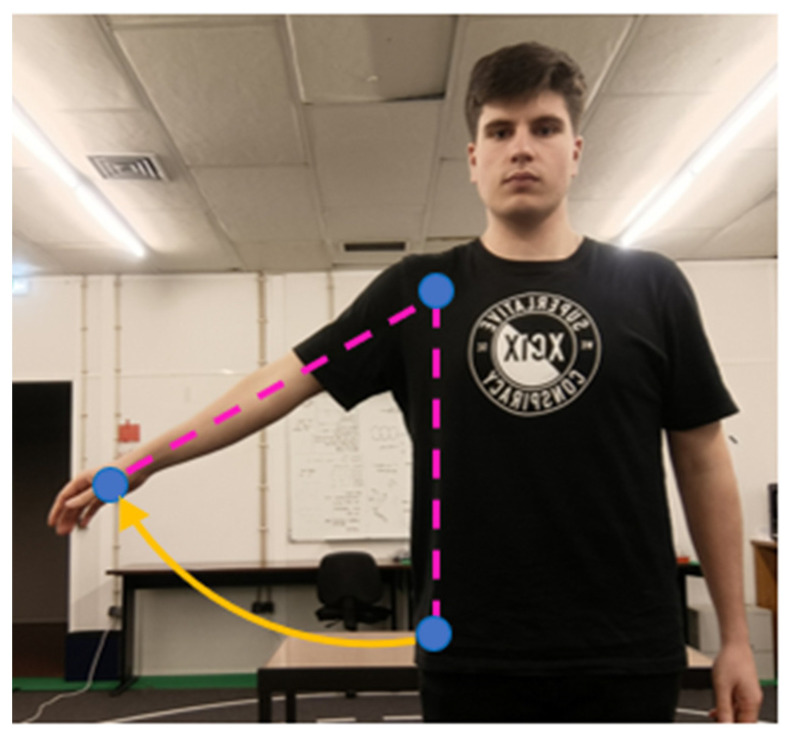
Representation of shoulder roll using frontal projection (x–y plane).

Shoulder roll captures the lateral elevation or depression of the upper arm, occurring in the frontal plane. As with shoulder pitch, the same anatomical reference points are used: hip, shoulder, and elbow. In this case, only the x and y coordinates are considered, as the motion lies within the frontal (x–y) plane. The corresponding geometric configuration is presented in Figure 6.

A known limitation of this method arises when the elbow is positioned significantly forward relative to the shoulder. Under these circumstances, the scalar product may yield an incorrect value due to the influence of depth disparity. To compensate for this, a corrective factor based on the difference in depth (z) between shoulder and elbow is introduced. The adjusted shoulder roll is given by:

(2) $${SR}_{adj}=SR\left(1-\frac{SD-ED}{20}\right),$$

where *SR* is the ShoulderRoll, *SD* is the ShoulderDepth and *ED* is the ElbowDepth.

This correction ensures a more accurate estimation by attenuating the roll value proportionally to the depth discrepancy.

Elbow Roll Calculation

**Figure 7:**
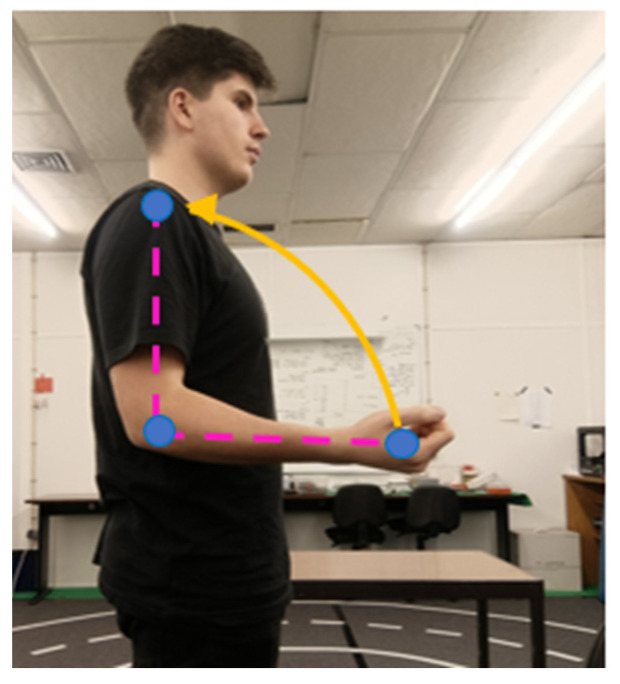
Estimation of elbow roll from shoulder–elbow–wrist vectors.

Elbow roll corresponds to the flexion-extension movement of the forearm, i.e., the opening and closing of the elbow joint. To calculate this angle, the scalar product is applied to vectors defined by the shoulder, elbow, and wrist points. The resulting configuration is shown in Figure 7.

Elbow Yaw Calculation

**Figure 8:**
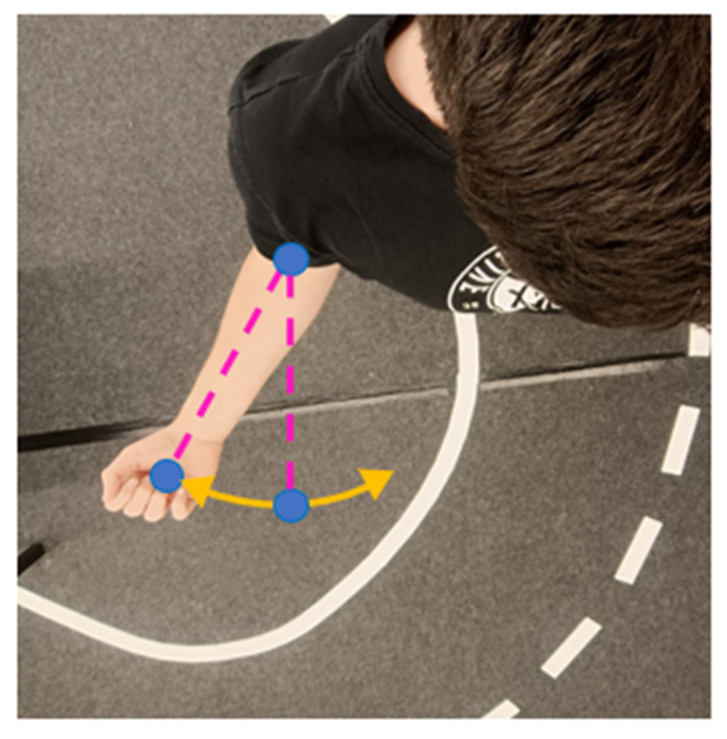
Elbow yaw estimation using auxiliary projection plane.

Elbow yaw defines the axial rotation of the forearm around the upper arm’s longitudinal axis, a motion that is inherently more difficult to capture via image-based estimation. The method employed here uses three points: the wrist, the elbow, and an auxiliary projection point that shares the x-coordinate of the elbow and the z-coordinate of the wrist, forming a reference plane for yaw detection (see Figure 8).

**Figure 9:**
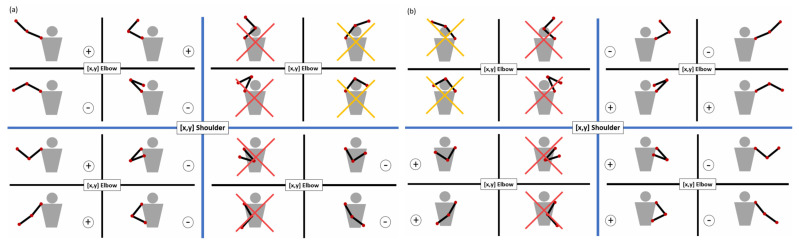
Classification of elbow yaw configurations: (**a**) right arm; (**b**) left arm.

A detailed classification of elbow torsion was conducted, analyzing combinations of relative positions of the shoulder, elbow, and wrist. This results in 16 possible positional scenarios: 4 combinations of elbow coordinates relative to the shoulder (based on x and y), each subdivided into 4 possible wrist positions relative to the elbow. These configurations are summarized in Figure 9, where subfigure Figure 9a depicts the right arm and subfigure Figure 9b the left arm. Red crosses indicate physically impossible poses, while yellow crosses represent poses that exceed the robot’s mechanical limits.

Wrist Yaw Calculation

Wrist yaw refers to the axial rotation of the hand relative to the forearm. Accurately estimating this degree of freedom using vision-based methods alone remains a challenge, particularly due to occlusions and the limited resolution of angular rotation from 2D projections. To address this, a geometric method was implemented based on the slope of two lines converging at the wrist joint. The first line connects the wrist to the metacarpophalangeal (MCP) joint of the index finger, and the second connects the wrist to the MCP joint of the pinky finger.

The slope m of a line defined by two points $\left(x_{1},y_{1}\right)$ and $\left(x_{2},y_{2}\right)$ is calculated as:

(3) $$m=\frac{y_{2}-y_{1}}{x_{2}-x_{1}},$$

Once the slopes $m_{1}$ and $m_{2}$ are obtained for both lines, the angle θ between them is computed using the arctangent of the difference in slopes:

(4) $$\theta =tan^{-1}\left[\frac{m_{1}-m_{2}}{1+m_{1}\cdot m_{2}}\right],$$

**Figure 10:**
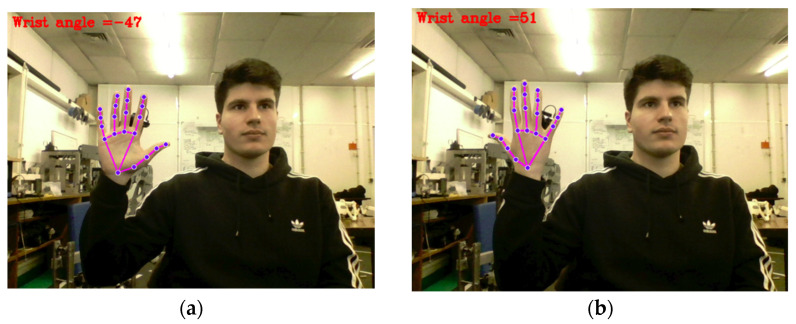
Estimation of wrist yaw: (**a**) θ=−47˚; (**b**) θ=51˚.

This angular difference represents the wrist yaw estimation and is visually demonstrated in Figure 10, where two wrist postures are compared under this method.

**Figure 11:**
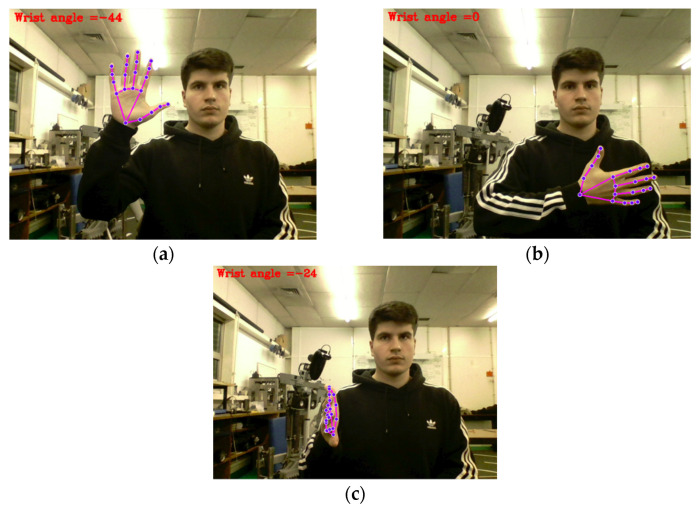
Wrist yaw estimation failure cases due to hand orientation: (**a**) palm fully visible and facing the camera; (**b**) palm facing away, with the back of the hand toward the camera; (**c**) palm partially rotated at an oblique angle.

While effective in many frontal-facing postures, this method exhibits limitations in situations where the hand is rotated toward the camera, causing the palm to be partially or completely occluded. In such cases, landmark visibility is reduced, and slope-based estimation becomes unreliable. This limitation is illustrated in Figure 11, which presents three distinct arm orientations. Despite the wrist being static in all instances, the calculated angles vary considerably due to projection distortions.

To overcome these challenges, an alternative strategy involves integrating Inertial Measurement Units (IMUs). These sensors provide direct gyroscopic readings of angular velocity and orientation, enabling accurate wrist rotation tracking even under conditions of occlusion or limited camera perspective.

Limitations of Vision-Based Axial Rotation Estimation

Estimating axial joint rotations, such as elbow yaw and wrist yaw, using vision-only pose estimation remains a well-known challenge in human–robot interaction systems. Unlike flexion–extension movements, axial rotations are particularly sensitive to occlusions, viewpoint changes, and landmark projection ambiguities, especially when relying on monocular or RGB-D cameras.

In the proposed system, elbow yaw estimation is performed using geometric relationships between the shoulder, elbow, and wrist landmarks. A systematic analysis of possible arm configurations was conducted, considering the relative positions of these joints in the image plane and depth axis. This analysis results in sixteen theoretical elbow torsion configurations, from which physically impossible poses and configurations outside the robot’s mechanical range can be explicitly identified. While this strategy improves robustness compared to naïve projection-based methods, it remains sensitive to self-occlusions and extreme viewpoints.

Wrist yaw estimation presents even greater limitations. The adopted method relies on the relative orientation of finger landmarks with respect to the wrist joint. Although effective when the palm is clearly visible to the camera, the estimation becomes unreliable when the hand rotates toward or away from the camera, leading to landmark overlap or loss of depth discrimination. In such cases, similar wrist orientations may produce significantly different estimated angles, despite no actual rotation being performed.

Rather than attempting to mask these limitations, the system explicitly identifies operating conditions under which axial rotation estimates are reliable, partially reliable, or unreliable. In practice, wrist yaw estimation is considered reliable only when the hand orientation allows clear separation of finger landmarks, while elbow yaw estimation degrades primarily under strong occlusions or extreme arm postures.

No absolute angular error metric is computed for axial rotations, as ground-truth joint orientations were not instrumented during experiments. Instead, reliability is assessed at the task level, focusing on the successful execution of manipulation actions. These findings motivate the integration of complementary sensing modalities, such as IMUs, which are discussed as a concrete extension in future work.

Hand Opening and Closing

A fundamental capability in teleoperation is the ability to grasp and release objects. Although this system does not implement full individual finger articulation, it supports binary hand states: open and closed. Detection is based on the distance between the wrist and the tip of the middle finger, computed in 3D space using the Euclidean distance formula:

(5) $$dist=\sqrt{(W_{x}-M\_tip_{x}{)}^{2}+(W_{y}-M\_tip_{y}{)}^{2}+(W_{z}-M\_tip_{z}{)}^{2}},$$

where *W* is the wrist landmark and *M* corresponds to the middle fingertip. This metric allows a threshold-based approach to infer whether the hand is open (large distance) or closed (short distance), enabling basic grasp control for object manipulation in remote robotic operation.

## 4. Virtual Reality Telepresence Interface

Telepresence technologies enable users to remotely control and interact with robotic systems located in distant or inaccessible environments. When integrated with VR, these systems provide immersive experiences that enhance spatial awareness and control fidelity, allowing users to perceive and respond to the remote environment as though they were physically present.

For this system, a PX2.0 virtual reality headset was employed due to its affordability and satisfactory immersive performance. The headset features adjustable lenses and is designed to house a smartphone that displays two separate images, one for each eye, creating the stereoscopic effect required for VR. The visual feed is processed using OpenCV and delivered to the smartphone via a web interface, enabling real-time rendering of the robot’s field of view.

**Figure 12:**
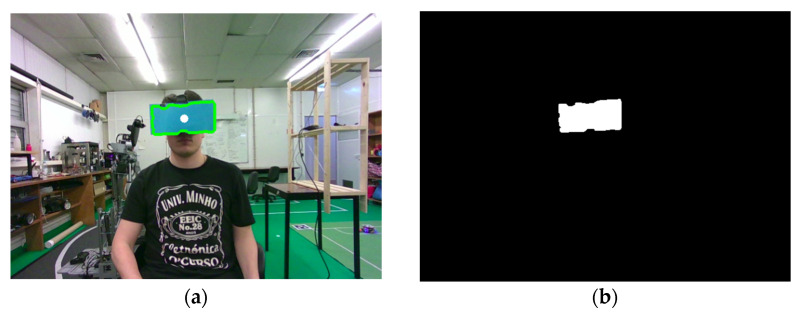
Blue color contour and centroid detection (**a**) with corresponding HSV (Hue–Saturation–Value) mask (**b**).

During implementation, a key challenge emerged: standard facial detection and head pose estimation algorithms failed when the headset was worn, as the user’s face was obscured. To resolve this, a colored surface was affixed to the front of the headset, and an HSV (Hue–Saturation–Value) mask was applied to isolate that color. Contour detection was then performed on the mask, and the centroid of the detected region was computed. This point served as an approximate proxy for the nose position. The process is shown in Figure 12, with the original image and corresponding binary mask.

After identifying the centroid, depth data were retrieved to compute the 3D position of the detected point. To calculate the head pitch angle, two additional reference points were constructed: one located centrally along the shoulder line, and a second positioned perpendicularly at half the shoulder width, maintaining the same depth. These three points form a triangle in the sagittal plane, allowing for the estimation of head inclination.

Nevertheless, the method exhibits inherent limitations. Its performance depends on consistent lighting conditions and adequate color contrast between the patch and the surrounding environment. Strong illumination changes, shadows, or the presence of similar colors in the background may degrade segmentation accuracy, leading to noisy or unstable head pose estimates. These limitations are intrinsic to color-based vision methods and were considered acceptable within the scope of a low-cost, prototype-oriented teleoperation framework. This solution is not intended as a general replacement for model-based or sensor-based head tracking. Instead, it serves as a lightweight workaround that enables head pose control in scenarios where facial landmark detection is not feasible. For applications requiring higher robustness or operation in uncontrolled environments, the integration of IMUs is identified as a natural path to follow.

**Figure 13:**
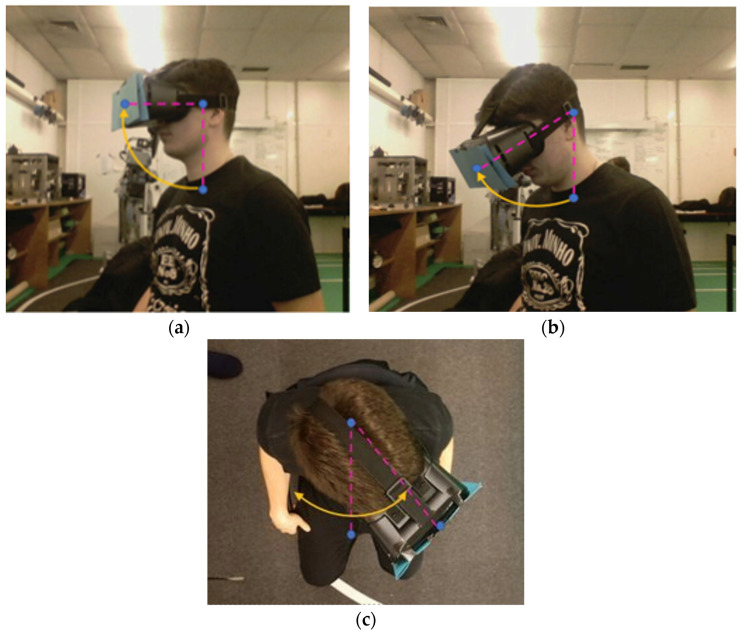
Estimation of head pose: (**a**) mask centroid and shoulder center, (**b**) shoulder width projection for pitch, (**c**) XZ-plane configuration for yaw angle.

For head yaw estimation, a method analogous to the elbow yaw procedure was adopted. The reference point above the shoulders was paired with another point sharing the same x and y coordinates but placed at a different depth along the *z*-axis. The angle between these two vectors and the mask centroid in the XZ (top-down) plane yielded the yaw measurement. This process is illustrated in Figure 13, where subfigures Figure 13a,b depict the pitch estimation geometry, and Figure 13c shows the yaw estimation setup.

## 5. Experiments and Results

Teleoperating a robot equipped with an anthropomorphic arm introduces both opportunities and challenges. On one hand, this configuration enables dexterous manipulation; on the other, it imposes strict requirements for low-latency and accurate communication between the operator and the robot. Two experimental setups were conducted to evaluate the system’s functionality: one for direct control of the robotic arm, and another for full VR-based teleoperation.

Controlling the Anthropomorphic Arm

**Figure 14:**
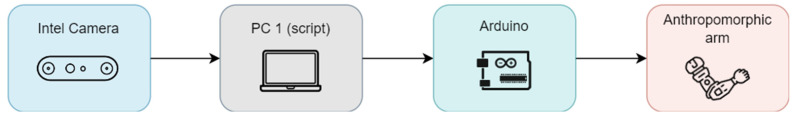
Communication architecture during anthropomorphic arm control test.

The initial test focused on evaluating the operation of CHARMIE’s robotic arm [17]. Custom modifications were applied to the communication interface with the Arduino Mega, which handles motor actuation. As illustrated in Figure 14, the system architecture consists of an Intel RealSense camera, a processing unit (PC1), the Arduino board, and the robotic arm itself.

Given the continuous variation in joint angle values, directly applying these values to the servos can result in erratic or unstable movement. To mitigate this, a filtering mechanism was introduced: a cyclic buffer accumulates recent values, and an averaging filter ensures smooth transitions. Additionally, calculated joint angles may exceed the physical range of the servos. To avoid damage or loss of accuracy, a mapping function interpolates the computed angle to match the servo’s operational limits. This is defined as:

(6) $$Servo\_value=max\left\{\begin{array}{c} min\left\{\frac{\begin{array}{c} {out}_{max}\\ \left(x-{in}_{min})\cdot ({out}_{max}-{out}_{min}\right) \end{array}}{{in}_{max}-{in}_{min}}\right.+{out}_{min}\\ {out}_{min} \end{array}\right.,$$

where *x* is the calculated joint angle, $in_{min}$ and $in_{max}$ define the expected input range, $out_{min}$ and $out_{max}$, denote the physical servo limits.

**Figure 15:**
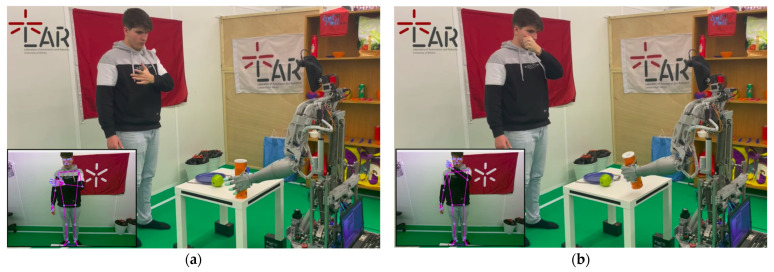
Object manipulation test with anthropomorphic arm: (**a**) grasping; (**b**) lifting.

To validate the system, the RealSense camera was mounted on CHARMIE’s head, providing real-time visual data. The operator stood in front of the robot and performed object manipulation tasks. The results, shown in Figure 15, confirm successful grasping and controlled movement of the target object.

Across repeated trials, the proposed mapping enabled consistent execution of reaching and grasping motions without loss of object stability. Task completion was achieved whenever the target object remained within the camera field of view and axial wrist rotation was not dominant, highlighting the practical reliability of the approach under typical operating conditions.

Experimental Evaluation Criteria

Given the system-oriented nature of the proposed teleoperation framework, experimental evaluation was conducted at the task level rather than through per-joint angular error analysis. The primary objective was to assess whether the pose estimation and control pipeline enabled reliable and repeatable execution of manipulation tasks under realistic operating conditions.

Three qualitative–quantitative criteria were adopted:

- Task completion success, defined as the robot’s ability to reach, grasp, and lift a target object without external intervention.
- Execution consistency, assessed by observing repeatability of the task outcome across multiple trials under similar conditions.
- Operational stability, evaluated through the absence of abrupt or unsafe motions during teleoperation, particularly under continuous arm and head movements.

These criteria were selected to reflect practical performance in service robotics scenarios, where successful task execution and system stability are often more relevant than precise joint-level error metrics. While no ground-truth motion capture system was employed, the evaluation provides objective evidence of functional teleoperation capability.

Full Robot Teleoperation via Virtual Reality

The second test evaluated the complete teleoperation system, in which the operator controlled both the robot’s arm and head movements using VR. The operator received real-time visual feedback and used head movements to direct CHARMIE’s gaze. Simultaneously, arm gestures were mapped to the robotic arm, enabling coordinated and intuitive control.

**Figure 16:**
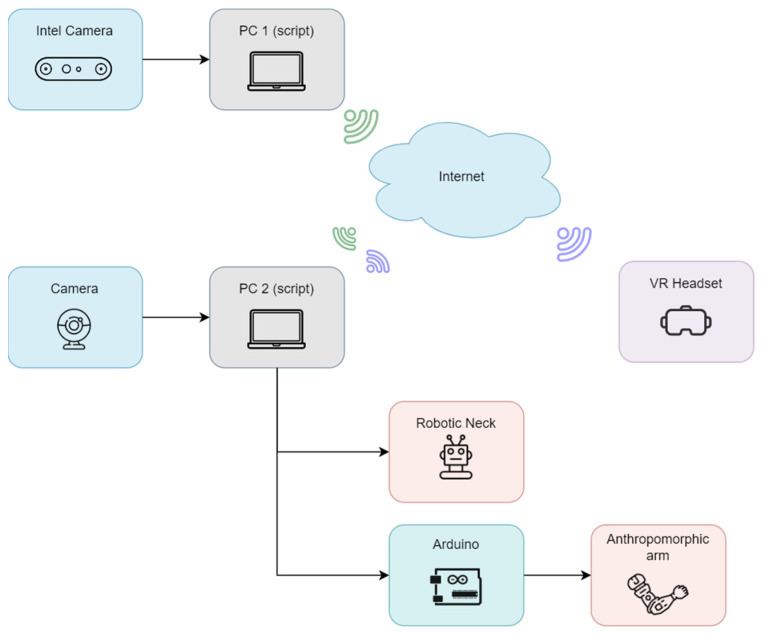
Communication architecture for VR-based full robot teleoperation.

Figure 16 shows the system architecture. The Intel camera captures the operator’s movements and sends data to PC1, where joint angles are computed. These values are transmitted via socket communication to PC2, which handles robotic actuation. PC2 also receives video streams from CHARMIE’s onboard camera and performs stereoscopic rendering for the VR headset, allowing the operator to perceive the environment immersively.

During VR-based teleoperation, stable control was achieved for most arm movements and object manipulation actions. However, reduced stability was observed during axial wrist rotations under unfavorable viewpoints, particularly when hand landmarks became partially occluded. This behavior is consistent with the known constraints of vision-only yaw estimation, as discussed earlier.

**Figure 17:**
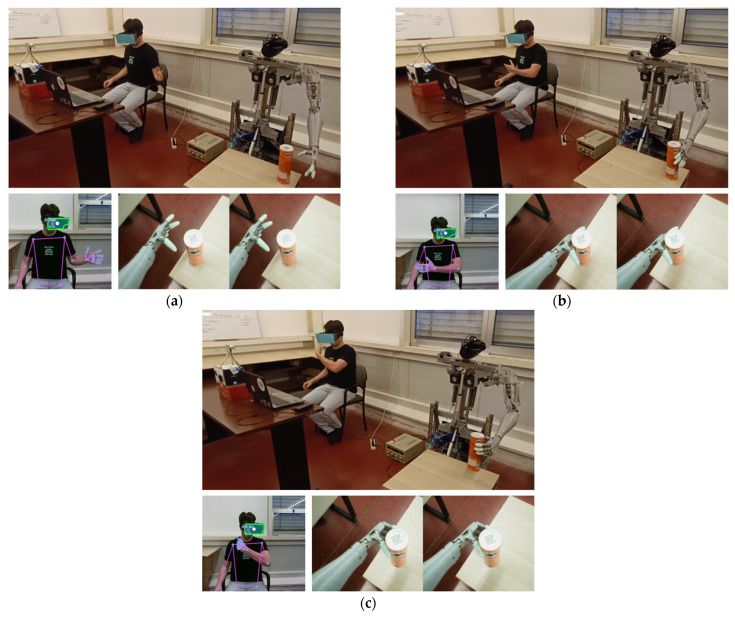
Object manipulation via VR teleoperation: (**a**) reaching for the object, (**b**) establishing contact, and (**c**) successful grasp.

The outcomes are presented in Figure 17, which displays three key moments from the robot’s perspective. In subfigure Figure 17a, the arm reaches toward the object; in Figure 17b, contact is established; and in Figure 17c, the object is successfully grasped. Across repeated trials, the robot successfully completed the reach–grasp–lift sequence without loss of object stability, demonstrating consistent mapping between human arm motion and robotic actuation. These results demonstrate the system’s capacity for precise and responsive manipulation under VR-based teleoperation.

Discussion and Comparison with Related Work

When compared with existing VR-based teleoperation approaches reported in the literature [9,10,11,12], several distinctions can be highlighted. Many existing systems prioritize immersive interaction within virtual or simulated environments, often without validation on physical robotic platforms or under real-world operational constraints. Vision-based teleoperation approaches demonstrate promising results but frequently provide limited analysis of pose estimation reliability, particularly under occlusions and axial joint rotations.

In contrast, the present work combines RGB-D sensing with immersive VR feedback and validates the complete pipeline on a real service robot performing object manipulation tasks. Rather than assuming ideal pose reconstruction, this study explicitly analyzes the limitations of vision-only estimation and evaluates performance at the task level. This positioning reflects a pragmatic trade-off between system complexity, cost, and functional robustness, which is particularly relevant for domestic and service robotics applications.

## 6. Conclusions and Future Work

This study presents a teleoperation framework for the CHARMIE service robot, integrating 3D human pose estimation and a virtual reality interface to enable intuitive and immersive remote control. The system demonstrated its effectiveness in real-world scenarios involving object manipulation, confirming the feasibility of vision-based pose tracking for human–robot interaction.

Experimental evaluation demonstrated that the proposed system enables reliable task-level teleoperation, particularly for reaching and grasping actions, without requiring instrumented environments or wearable sensors. While axial wrist rotations remain challenging under vision-only sensing, successful task execution was consistently achieved under typical operating conditions, validating the feasibility of the proposed low-cost teleoperation framework.

Despite the promising results, the experiments also revealed limitations inherent to vision-only approaches, particularly in estimating wrist and elbow axial rotations. Inaccuracies in Wrist Yaw estimation were noted, especially under partial occlusions or when the hand faced away from the camera, as discussed in previous sections. These findings indicate the need for complementary sensing modalities to enhance robustness.

Although the proposed system relies exclusively on vision-based sensing and visual feedback, it does not currently incorporate force or haptic feedback. Bidirectional force control can enhance immersion and precision in teleoperation, particularly in contact-rich manipulation tasks. However, implementing force feedback requires dedicated hardware, accurate force sensing at the robot side, and stable bilateral control strategies to ensure safety and transparency. These requirements substantially increase system complexity and cost, which contrasts with the primary objective of this work: to develop a low-cost, lightweight, and easily deployable teleoperation framework based on vision and VR. For these reasons, force feedback was intentionally excluded from the current implementation and is considered a natural extension for future work.

Future development of the system will focus on the following directions:

- Enhanced rotational tracking through the integration of IMUs, particularly to improve the estimation of Wrist Yaw and Elbow Yaw angles.
- Utilization of smartphone gyroscopes embedded in the VR headset to achieve more precise head pose estimation and improve immersive control.
- Design of an operator interface that overlays real-time robot feedback directly into the VR environment, improving situational awareness and control usability.
- Deployment of a tactile sensing layer on the robot’s forearm to detect contact and interaction forces in real time, thereby improving safety and enabling haptic feedback during manipulation tasks [18].

While the current mapping algorithm provides satisfactory results for basic teleoperation, discrepancies between human and robot movements were observed, particularly during more complex joint configurations. As visible in Figure 16, mismatches between the operator’s intended motion and the robot’s execution highlight the need for refinement.

To address these issues, future work will explore dynamic calibration and adaptive filtering techniques, taking into account both the robot’s mechanical constraints and real-time sensor feedback. Additionally, learning-based methods—such as neural networks or regression models—will be investigated to continuously optimize the mapping function. Incorporating closed-loop feedback from proprioceptive and tactile sensors may also allow the system to self-correct motion discrepancies during execution.

Altogether, these enhancements are expected to advance the system toward a more reliable, flexible, and immersive teleoperation solution, facilitating its adoption in complex service robotics applications across diverse domains.

## Figures and Tables

**Table 1:** Technical specifications of the Intel^®^ RealSense™ D455 and Microsoft Kinect for Xbox 360.

	Intel^®^ RealSense™ D455	Microsoft Kinect XBOX 360
Dimensions	124 × 26 × 29 mm	249 × 66 × 67 mm
Recommended Range	40 cm to 6 m	1.2 m to 3.5 m
Minimum depth	40 cm	50 cm
Maximum depth	20 m	5 m
Accuracy range	Up to 1%	Up to 4%
Depth resolution	Up to 1280 × 800	640 × 480
Depth frame rate	Up to 90 fps	30 fps
Depth FOV	86° × 57° (±3)	43° × 57° (±27)
RGB resolution	Up to 1280 × 800	640 × 480
RGB frame rate	Up to 90 fps	30 fps
RGB FOV	86° × 57° (±3)	43° × 57° (±27)

## Data Availability

The data presented in this study are available on reasonable request from the corresponding author.
